# A call for action: Guidelines for implementation of mental health services at the community level

**DOI:** 10.4102/hsag.v31i0.3287

**Published:** 2026-08-31

**Authors:** Khalaeng F. Thobane, Fhumulani M. Mulaudzi, Ndivhaleni R. Lavhelani, Rodwell Gundo

**Affiliations:** 1 Department of Nursing Sciences, Faculty of Health Sciences, University of Pretoria, Pretoria, South Africa

**Keywords:** content, guideline, implementation, mental health services, community health workers

## Abstract

**Background:**

Mental health challenges account for approximately 18% of global disease burden. Few people with mental health challenges receive the required treatment, especially at community level. Implementation of mental health services at the community level is vital.

**Aim:**

The study aimed to explore and describe the content for inclusion in the guidelines for the implementation of mental health services in the Ward-Based Primary Health Care Outreach Teams (WBPHCOTs) services in South Africa.

**Setting:**

Data was collected in subdistrict one offices from the experts in mental health services and WBPHCOTs working in Tshwane District, Gauteng province, South Africa.

**Methods:**

A qualitative, explorative and descriptive design that followed a Nominal Group Technique (NGT) for data collection. Eleven experts were purposively sampled for participation in the study. The NGT process was followed: silent generation of ideas, round robin, group discussion and clarification process. Finally, ranking of ideas and group consensus was reached.

**Results:**

The following content was identified for inclusion in the guidelines: visits to households, collaboration with mental health key stakeholders, training, mental health prevention and promotion, adherence to treatment and advocacy, mental health professionals’ support, and strengthening community mental health services.

**Conclusion:**

The study assisted in identifying the content to be included in the guidelines for the implementation of mental health services in the WBPHCOTs’ services.

**Contribution:**

This study provides the content that could be included in the development of the guidelines for the implementation of mental health services in the WBPHCOTs services, with the anticipated contribution to the improvement in the implementation of mental health services at community level.

## Introduction

Even though legislation and policies are available to implement mental health services at community level, the mental health services continue to be inadequate at community level. In response to the above, the World Health Organization (WHO) developed the optimum mix of services for mental health services, recommending the importance of community mental health services (Rosen, Gill & Salvador-Carulla 2020; WHO 2022). In support, Xiang et al. (2020) discovered that the high demand for the services of the specialised psychiatric hospitals is compounded by the lack of community mental health services. The finding revealed that the provision of mental health services at the community level still has some challenges that requires to be addressed to reduce the treatment gap.

The study conducted in the United States concluded that Community Health Workers (CHWs) have the potential to address mental health treatment gaps at community level, given that they are the trusted members of the communities they serve (Barnett et al. 2021). Similarly, the study by Asher et al. (2021) in Ethiopia revealed that it is possible to train the CHWs with the necessary skills to deliver services for people with mental health challenges in community-based settings. In support of the above, Mhlongo, Lutge and Adepeju (2020) acknowledged that the CHWs have a role to play in strengthening community healthcare (inclusive of mental healthcare, especially in Low- and Middle-Income Countries [LMICs], such as South Africa).

In support, Palomin et al. (2023) revealed that there are limited mental health professionals in LMICs such as South Africa, which resulted in a severe shortage of mental health professionals in the community, whereas the number of people suffering from mental health conditions is increasing. The above findings suggest that most of the people suffering from mental health conditions cannot access such services, especially at the community level. In response to the above, South Africa has developed the Ward-Based Primary Health Care Outreach Teams (WBPHCOTs) Policy Framework and Strategy as part of primary healthcare (PHC) reengineering programme, to improve community outreach initiatives (Schneider et al. 2020). The CHWs are appointed in the WBPHCOTs supervised by Outreach Team Leaders (OTLs) who are enrolled and professional nurses (Babalola et al. 2023).

The study by Kawaya (2023) explained that in South Africa, CHWs are lay healthcare workers who work in the health services and understand the language and culture of the community. In addition, Mhlongo et al. (2020) revealed that CHWs serve as the cornerstone of the Brazilian healthcare system, and South Africa has currently adopted this concept. According to Ramukumba (2020), the CHWs could ideally be responsible for duties such as home visits and registrations, patient referrals for medical consultation, health education and promotion, and promoting treatment compliance for individuals with chronic diseases.

Kawaya (2023) further clarified that the CHWs have also received standardised job-related training, which is shorter than that of health professionals, and their main objective is to provide the community with culturally appropriate health services. The findings by Asher et al. (2021) and Kawaya (2023) reveal that the CHWs will require support and training from the mental health coordinators and professionals to enable them to implement the mental health services at community level. Furthermore, the WHO promoted the inclusion of CHWs in the provision of mental health services in a community-based setting (Barnett et al. 2018).

In contrary, a survey conducted in South Africa revealed that 64.8% of CHWs did not feel secure enough to care for or speak to people who had mental health challenges (Nyalunga et al. 2019). However, Sibeko et al. (2018) discovered that the CHW training intervention led to improvements in knowledge and confidence in supporting mentally ill patients. The findings imply that with training provided, the CHWs should be able to provide the community mental health services through the outreach programmes that they are conducting. There are policies that support the integration of mental health services into the WBPHCOT’s services.

Despite the recognised role of CHWs and existence of policies, there is a paucity of evidence on how mental health services are effectively implemented by the WBPHCOTs within South African context. Given the above, there is a need to develop guidelines for the implementation of mental health services in the services of the WBPHCOTs where the CHWs are working in the South African context. Therefore, the aim of this study was to explore and describe the content for inclusion in the guidelines for the implementation of the mental health services in the WBPHCOT’s services in South Africa using the Nominal Group Technique (NGT). This research is anticipated to contribute to the improvement in the implementation of mental health services at community level.

## Research methods and design

### Research approach

The study adopted a qualitative explorative, descriptive design whereby a NGT was utilised to reach consensus among a panel of experts from mental health professionals and WBPHCOTs professionals on the content to be included in the guidelines for the implementation of mental health services in the WBPHCOTs’ services. Nominal group technique involves a structured face-to-face meeting, typically involving 4–7 participants to reach a consensus on a subject matter (Humphrey-Murto et al. 2023). In this study, the main reason for choosing NGT was to rank the content and the literature of what was to be included in the guidelines and for a group of experts to reach consensus. Nominal group technique was utilised for the 11 experts to reach consensus on what content to include in the guidelines for the implementation of mental health services in the WBPHCOTs services.

### Study setting

The study setting is subdistrict one in the Tshwane District, Gauteng province, South Africa. The subdistrict one has a mixture of both peri-urban and urban townships. The subdistrict has a population of 811 570 (Madlala, Joubert & Mokoena 2025). The area has a high prevalence of substance abuse and common mental health conditions such as anxiety and depression (Mokwena & Madiga 2023). The reason for choosing sub-district one was that, according to the District Health Management Information System (DHMIS) for the year 2022 in Gauteng province, the sub-district had a higher number of patients who were taking mental health treatment compared to other sub-districts. The subdistrict health services are responsible for the rendering of primary health and community health services to the local communities.

### Study population and sampling strategy

#### Population

The population comprised of all the OTLs in the subdistrict one and the mental health coordinators in the Tshwane District. The OTLs are the registered or enrolled nurses appointed as OTLs in the WBPHCOTs, and they are responsible for supporting and managing the CHWs in the WBPHCOTs.

#### Sampling strategy

A purposive sampling method was utilised to identify 11 participants who participated in the study. Purposive sampling is a nonprobability sampling strategy in which the researcher chooses participants based on their subjective assessment of who is more knowledgeable about the study topic (Polit & Beck 2021). In this study, experts were invited using purposive sampling based on knowledge about WBPHCOTs and mental health services in the community. Nominal group research utilises groups of four to seven participants, where a limit of seven members has been recommended (McMillan, King & Tully 2016; Vahedian-Shahroodi et al. 2023).

The participants were divided into two groups, consisting of five and six members, respectively. The NGT 1 consisted of two mental health coordinators and three OTLs, while the NGT 2 had four mental health coordinators and two OTLs. In total, six mental health coordinators and five OTLs participated in the study. Consensus on what content to be included in the guidelines for implementation of mental health services in the WBPHCOT’s services was reached by both groups.

The inclusion criteria for the OTLs were the following: (1) registered nurses or enrolled nurses appointed as OTLs in the WBPHCOTs; (2) currently involved in the support and management of CHWs working in the WBPHCOTs; and (3) more than 5 years of work experience as a nurse. The inclusion criteria for mental health coordinators were the following: (1) be employed as a mental health coordinator in Tshwane Health District; and (2) have more than 5 years’ work experience. See Table 1 for the demographic data of the participants.

**TABLE 1:** Demographic data of 11 nominal group technique participants.

Criterion	Characteristics	Frequency		Percentage
		NGT 1	NGT 2	
Age	17–30 years	0	0	0.0
	31–45 years	3	0	27.3
	> 45 years	2	6	72.7
Race	White people	1	0	9.1
	African people	4	6	90.9
	Mixed race people	0	0	0.0
	Indian people	0	0	0.0
Gender	Males	1	2	27.3
	Females	4	4	72.7
Job title	Occupational therapist	1	0	9.1
	Professional nurses	2	4	54.5
	Enrolled nurses	2	2	36.4
Years of experience	6–15 years	3	1	36.4
	16–25 years	1	4	45.5
	> 25 years	1	1	18.1

*Source:* Adapted from, Wallace, S.J., Barnett, A., Cheng, B.B., Lowe, J., Campbell, K.L. & Young, A.M., 2023, ‘What is “successful rehabilitation”? A multi-stakeholder nominal group technique study to inform rehabilitation outcome measurement’, *Clinical Rehabilitation* 37(9), 1248–1259. https://doi.org/10.1177/02692155231157181

NGT, nominal group technique.

### Data collection

#### Invitation of participants

The participants were invited via email, with assistance from the subdistrict one mental health coordinator. An email was sent to the WBPHCOTs district manager to request the participation of the OTLs in the NGT discussions. Another email was sent to the district mental health manager to request the participation of the mental health coordinators in the NGT discussions. Then individual emails were sent to participants inviting them to participate in the study. The inclusion criteria were stated at the time of the email, and only those meeting the criteria were allowed to participate.

The two NGT sessions were held in a boardroom at the sub-district offices on separate days. Each session was held for a maximum of 2 h. As a professional nurse with years of experience in mental health, the researcher facilitated the NGT meetings. The notes were written, and an audio tape was utilised to record the sessions.

#### The nominal group technique process

The NGT was chosen as a method for exploring and describing the content that could be included in the guidelines for the implementation of mental health services in the WBPHCOTs’ services. The NGT is a practical method that is easy to employ to elicit individual opinions on the content to be included in the guidelines for the implementation of mental health services in the WBPHCOT’s services (Cooper et al. 2020). The NGT has been broadly applied to clinical or medical care (Jain et al. 2020). Nominal group technique eliminates various challenges that frequently arise in group meetings, such as strong personalities or deference to seniority, by guaranteeing that all points of view are given equal opportunity and that each group member has an equal chance to voice their thoughts (Ho et al. 2012; Vahedian-Shahroodi et al. 2023).

The NGT is a highly organised in-person group interaction that gives participants a voice and allows them to have their thoughts heard by other participants (McMillan et al. 2016; Vahedian-Shahroodi et al. 2023). Furthermore, McMillan et al. (2016); Vahedian-Shahroodi et al. (2023) described NGT as seeking to bring experts’ opinions on a certain subject into a consensus or agreement. The NGT was designed by Delbecq and Van de Ven and consists of four key stages: silent generation, round robin, clarification and voting (ranking or rating) (Delbecq, Van de Ven & Gustafson 1975; Vahedian-Shahroodi et al. 2023), as depicted in Figure 1:

**FIGURE 1:**
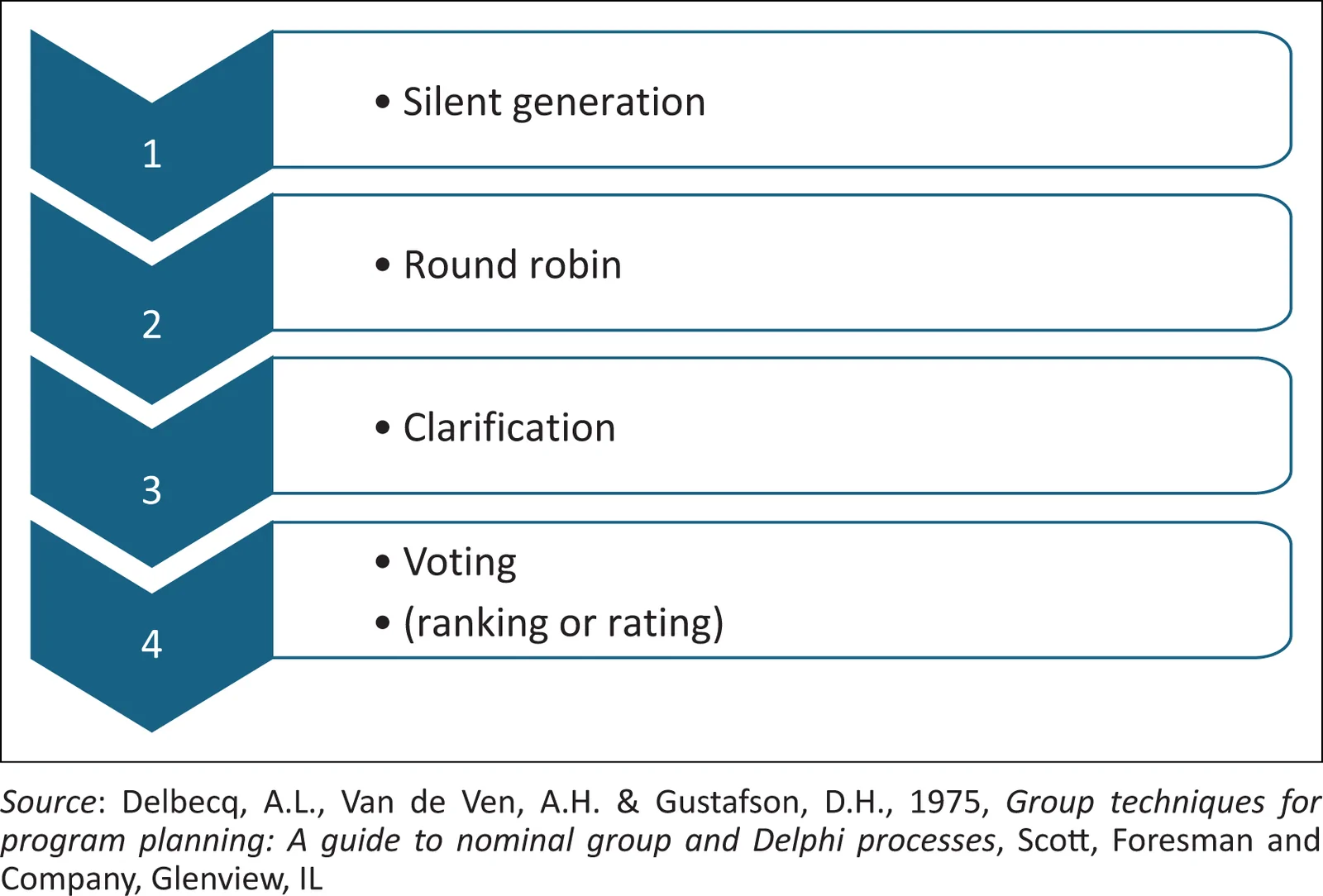
A simplified model of the nominal group technique process.

Two subsequent NGT discussions were conducted in March 2025. The researcher facilitated the NGT discussions. All participants were informed about the objectives of the study and how the study results will be utilised. The participants’ anonymity was guaranteed by the researcher. The two NGT group discussions were responding to the same question. Questions asked during the NGT:

- *What should be included in the guidelines for the implementation of mental health services in the WBPHCOT services?*

Before the start of the NGT session, the researcher gave a brief introduction on the context of the study topic, which is the implementation of mental health services in the WBPHCOT’s services, and on the purpose of the exercise. No hints were given by the researcher on the intended material of the responses. Participants were allowed to structure their responses in any format they wanted. At the start of the NGT, the researcher just presented the research question. Before the start of the NGT, participants were asked to provide recommendations on what should be included in the guidelines for the implementation of mental health services in the WBPHCOTs’ services. Both NGT group sessions consisted of four steps as outlined by Delbecq et al. (1975); Vahedian-Shahroodi et al. (2023):

**Step 1: Silent generation of ideas:** At the beginning of both NGT sessions, the participants were given 20 min to silently reflect and write down their ideas individually in response to a question that was posed to them by the researcher. The participants were requested to write down their ideas in bullet points. Participants were not interacting at this point (Vahedian-Shahroodi et al. 2023).

**Step 2: Round robin:** The researcher asked one participant at a time to state a single idea to the group in a round robin fashion and recorded each idea concisely. Each participant contributed one idea at a time until all ideas were exhausted, and they had the opportunity to voice their opinions freely, without rejection or modification from other participants (Vahedian-Shahroodi et al. 2023). The researcher recorded the presented ideas on a flip chart. In both NGT sessions, a total of 50 ideas were recorded. The participants were given 15 min for the task.

**Step 3: Clarification:** The participants were invited to clarify and elaborate on the ideas that they produced during the round robin step. The purpose of this step was to ensure that all listed ideas are clearly understood, to enable the facilitation of the voting or ranking in Step 4. All the participants contributed to the discussion and explained their ideas for engagement. Based on an open discussion between the participants, where there was no value judgement or ranking of ideas, similar ideas were grouped (Vahedian-Shahroodi et al. 2023). Following the grouping of similar ideas in both NGT sessions, the reconciliation of the ideas was reduced from 50 to 7. Fifteen minutes were allocated for clarification.

**Step 4: Voting and ranking:** The participants were asked to rate each of the mentioned ideas in a public vote in this step. To evaluate the level of acceptance for the suggested ideas, participants were invited to cast their votes in an open and non-anonymous manner. In the two NGT groups, each participant received five votes, enabling them to choose five ‘inspiring’ characteristics. The first NGT session resulted in 25 votes, while the second NGT session resulted in 30 votes. Therefore, the total votes received are 55. Individual participants were not allowed to cast more than one vote per idea. The researcher collated the voting results and displayed the number of votes for each suggestion on the flip chart (Vahedian-Shahroodi et al. 2023). The group’s favourite ideas were those with the highest ratings. Twenty minutes were allocated.

### Data analysis

Following an NGT in steps 2 and 3, where the group had synthesised the major concepts, the researcher already had a general understanding of the group’s views. The rating and voting phases in Step 4 then revealed the scores. This made it possible to convey the main findings, which were co-produced by the participants and presented in real time by the researcher to the participants right away. The researcher wrote the generated list on the board according to their priority and gave the participants a chance to examine the list for any changes. All participants agreed on the final ranking. The participants reached consensus on the content to be included in the guidelines for the implementation of mental health services in the WBPHCOTs’ services.

The researcher transcribed audio recordings manually into written transcripts, which were completed in a month following the data collection. The transcripts were repeatedly reviewed by the researcher to ensure accuracy.

Content analysis was employed to analyse data. As proposed by Bengtsson, the content analysis steps are as follows: decontextualisation, recontextualisation, categorisation and compilation (Bengtsson 2016).

Decontextualisation – the researcher in this study became acquainted with the data by reading the transcribed text to understand the full picture before breaking it up into smaller meaning units and giving each produced unit a code. Recontextualisation – the researcher compared the final meaning unit list with the original data to see if all the data were covered regarding the study’s objectives. Categorisation – the researcher created themes in this study after reading and comprehending the transcribed data. Compilation – the study’s researcher included a list that summarised the themes.

Content analysis was utilised to analyse and formulate the themes from the qualitative data that was presented by the participants. According to Kleinheksel et al. (2020) content analysis is the act of grouping texts into related categories while taking the participant and context into account to find patterns, relationships and similarities and contrasts that are both explicit and implicit within the text. Data were analysed manually. In developing the themes, the voted ideas were grouped into similar meanings to ensure that the voted ideas of the participants were represented.

### Measures of trustworthiness

The five-framework outlined by Denzin and Lincoln (eds. 2005) was utilised to adhere to measures to assure reliability. According to Polit and Beck (2021), these criteria include authenticity, dependability, credibility, transferability and confirmability. Credibility was ensured through adhering to the NGT methods and procedures thoroughly, which included voting and reaching consensus. The participant’s verbatim quotes were utilised to support the ideas that were generated and discussed to develop the themes and subthemes. Audio-recordings were utilised for data collection and documented transcripts to achieve triangulation.

Transferability demonstrates the extent to which the research findings can be applied to different contexts, individuals, groups and environments (Kakar et al. 2023). In this study, transferability was ensured by using purposive sampling to select the participants who work in mental health and WBPHCOTs to provide rich descriptions and share their experiences about the topic. To guarantee dependability, the researcher employed a dependability audit and stepwise replication code recoding of the data. To ensure that the researcher considers their own prejudices and to prevent contaminating the study results, bracketing and field notes were utilised (Polit & Beck 2021). Therefore, reflexivity ensured the study’s confirmability. The researcher reflected the opinions, viewpoints, assertions, concerns and voices of each study participant; therefore, authenticity was guaranteed.

### Ethical considerations

This study received ethical approval from the University of Pretoria’s research committee, and the reference number is 403/2023. In addition, approval from the Gauteng Department of Health ethics committee was received with reference number GP_202308_015. Finally, permission to enter the research site was granted by the area and programme managers.

## Results

### Demographic characteristics of participants

A total of 11 participants participated in the NGT process and were included in the data analysis. The participants for both groups consisted of 1 (9.1%) Occupational Therapists, 6 (54.5%) Professional Nurses and 4 (36.4%) Enrolled Nurses. A total of 3 (27.3%) males and 8 (72.7%) females participated in the study. A total of 3 (27.3%) participants’ ages were between 31 and 45, while 8 (72.7%) were over 45 years. The participants had different years of work experience outlined as follows: 4 (36.4%) had 6–15 years, 5 (45.5%) had 16–25 years, and 2 (18.1%) had over 25 years. The demographic data are depicted in Table 1.

### Content recommended during the nominal group technique discussions

The seven contents that were recommended by the participants of the two NGT sessions for inclusion in the guidelines for the implementation of mental health services in the WBPHCOTs services are depicted in Table 2.

**TABLE 2:** Content to be included in the guidelines for the implementation of mental health services in the Ward-Based Primary Health Care Outreach Teams’ services.

Recommended content	NGT 1		NGT 2		Number of votes
	Votes	Ranking	Votes	Ranking	
Visits to MHCUs’ households	5	1	6	1	11
Collaboration of WBPHCOTs with mental health key stakeholders	5	1	6	1	11
Training of CHWs working in the WBPHCOTs	4	2	5	2	9
Mental health prevention and promotion	4	2	5	2	9
Adherence to treatment and advocacy for MHCUs	3	3	5	2	8
Mental health professionals’ support to WBPHCOTs	3	3	1	4	4
Strengthen community mental health services	1	4	2	3	3

*Source:* Adapted from, Kearney, N., Connolly, D., Bahramian, K. & Guinan, E., 2025, ‘Strategies to improve participation in exercise programmes during chemotherapy: A modified nominal group technique’, *Journal of Cancer Survivorship* 1–11. https://doi.org/10.1007/s11764-025-01771-y

NGT, nominal group technique; WBPHCOT, ward-based primary healthcare outreach team; CHW, community health worker; MHCUs, mental health care user’s.

The number of votes represented the desire for that content to be included in the guidelines. Therefore, the content with the higher number of votes was viewed by the participants as the most significant and was ranked high. The seven recommended contents were presented according to their level of importance as per the participants’ votes and were listed as follows: visits to mental health care user’s (MHCU) households (11 scores), collaboration of WBPHCOTs with mental health key stakeholders (11 scores), training of CHWs working in the WBPHCOTs (9 scores), mental health prevention and promotion (9 scores), adherence to treatment and advocacy for MHCUs (8 scores), mental health professionals’ support to WBPHCOTs (4 scores) and strengthen Community mental health services (3 scores). The seven recommended contents and the corresponding actions to guarantee their implementation are shown in Table 3.

**TABLE 3:** Recommended content and the action plans for the guidelines for the implementation of mental health services in the Ward-Based Primary Health Care Outreach Teams services at the community level.

Recommended content	Actions
Conducting home visits to MHCUs’ households by CHWs in WBPHCOT.	CHWs in the WBPHCOT could do the following: Conduct home visits to MHCUs’ households, which will assist in their recovery and treatment adherence. Encourage self-care of the MHCUs. Provide psychoeducation (according to their capacity) during home visits to the MHCU and the family. Have knowledge on how to reach OTLs and mental health care professionals when the CHWs are doing site visits for any form of support they might need. Conduct home visits in a manner that upholds dignity and confidentiality, promotes family participation in the care of their loved one, assesses the environment and social determinants, links the households to community support and respects cultural and spiritual beliefs.
Collaboration of WBPHCOTs with key mental health stakeholders.	WBPHCOT members could do the following: Participate in the mental health intersectoral forums where different stakeholders are involved for them to be familiar with the stakeholders. Attend scheduled meetings of the intersectoral forums. Compile a list of the available resources within their area to enable them to have a database of the available services. Maintain a collaborative relationship with the key stakeholders, inclusive of families, community leaders, traditional, social development and faith-based organisations, acknowledging their interconnectedness.
Training of Community Health Workers (CHWs) in the early identification of mental health conditions, screening, and referral to PHC clinics.	The Department of Health could do the following: Train CHWs on screening of common mental health conditions and referral pathways. Train CHWs to be able to identify cases that need immediate intervention and refer or report to relevant key stakeholders. Train CHW’s immediate supervisors, being the Outreach Team Leaders (OTLs), on mental health so that they can provide support and guidance. Develop a training programme inclusive of mental health at the beginning of each financial year. Accredit the formal training of the CHWs with a curriculum inclusive of mental health. Provide the training that will equip the CHWs and OTLs with skills in person-centred care, family involvement, community participation and culturally or spiritually sensitive practices.
Participation of WBPHCOT in mental health prevention and promotion activities	CHWs (trained) in the WBPHCOT could do the following: Provide mental health education to the MHCUs and families during the household visit. Provide mental health information to the community during community meetings. Assist in screening for mental health and refer those with positive signs for treatment. Be able to identify signs of relapse and refer for further care, treatment and rehabilitation. Assist in conducting mental health awareness programmes in the community to reduce stigma. Participate in the mental health awareness activities programmes. Be involved in communicating mental health messages through platforms such as social media, local radio stations, community meetings, mental health campaigns, etc. Implement the mental health promotion and prevention activities that enhance self-care, family support, foster community cohesion, manage environmental risk factors and incorporate culturally and spiritually appropriate approaches.
Adherence to treatment and advocacy for MHCUs	CHWs in the WBPHCOT could do the following: Assist in monitoring compliance with treatment during the household visit. Assist in tracing the defaulters to prevent relapse. Advocate for the MHCUs where necessary. Remind the user and the family about the date for the follow-up at the clinic.
Provision of technical support to the CHWs in the WBPHCOT by Mental Health care professionals.	Mental healthcare professionals could do the following: Provide technical support to the CHWs in the WBPHCOT. Conduct regular meetings with the WBPHCOT to discuss the issues and challenges that the WBPHCOT are experiencing during their daily work. Provide in-service training for the OTLs and CHWs on common mental health conditions, introduction to screening tools and how to use and score these tools, and referral pathways. Provide training on Screening, Brief Intervention and Referral to Treatment (SBIRT) to the WBPHCOT. Be accessible by OTLs when they encounter difficult cases, as they are the first contact of the CHWs, should they encounter any challenges. Work closely with OTLs as the immediate supervisors of the CHWs in the WBPHCOT. Provide continuous training and support that enhance respectful, family-inclusive, community-based care, and culturally and spiritually sensitive mental healthcare while dealing with the environment of social determinants of mental health.
Strengthen community mental health services	The Department of Health could do the following: Strengthening the mental health referral system between the PHC facilities and the WBPHCOT. Capacitating the Primary Health Care facilities to be able to manage the referred cases. Strengthening mental health services at the primary healthcare level, which is very critical for the WBPHCOT to be effective in implementing mental health services. Providing the PHC facilities with trained mental health professionals who can render mental health services at the PHC level. Strengthen community mental health services through the promotion of a person-centred approach, family involvement, enabling community participation and addressing social determinants of mental illness and integrating culturally and spiritually sensitive approaches.

*Source:* Adapted from, Kearney, N., Connolly, D., Bahramian, K. & Guinan, E., 2025, ‘Strategies to improve participation in exercise programmes during chemotherapy: A modified nominal group technique’, *Journal of Cancer Survivorship* 1–11. https://doi.org/10.1007/s11764-025-01771-y

CHW, community health worker; WBPHCOT, ward-based primary healthcare outreach team; OTL, outreach team leader; PHC, primary health care; MHCUs, mental health care user’s.

## Discussion

The study was aimed to explore and describe the content for inclusion in the guidelines for the implementation of mental health services in the WBPHCOT’s services in South Africa. Two Nominal group sessions were held, and seven contents that were recommended for inclusion in the guidelines were identified as follows: visits to MHCUs’ households, collaboration of WBPHCOTs with mental health key stakeholders, training of CHWs working in the WBPHCOTs, mental health prevention and promotion, adherence to treatment and advocacy for MHCUs, mental health professionals’ support to WBPHCOTs, and strengthen community mental health services as depicted in Table 3.

Both groups believed that visits to MHCUs’ households by the WBPHCOTs and collaboration of WBPHCOTs with mental health key stakeholders were significant, given that the two were ranked number 1. Training of CHWs working in the WBPHCOTs and mental health prevention and promotion were also recognised as critical by the participants, as they were ranked number 2. Finally, adherence to treatment and advocacy for MHCUs, mental health professionals’ support to WBPHCOTs, and strengthen community mental health services were also considered critical, as they were also ranked 3, 4 and 5, respectively.

The visit to the MHCUs’ households by the CHWs in the WBPHCOTs was reflected as a critical function that needs to be included in the guidelines. In support, Susanti, Octaviani and Nufush (2025) asserted that the CHWs in the WBPHCOTs are at the front line of community-based services, providing psychoeducation, assisting in the early detection of mental illnesses and monitoring medication adherence with the assistance of families via a schedule of regular home visits. In addition, Mistry, Harris and Harris (2021) explained that the outreach door-to-door visits by CHWs include a variety of mental health services such as, monitoring of MHCU’s selfcare, mental health education to the MHCU and the family, monitoring of the signs of relapse, and referral to the clinic for any challenges that may arise. Mhlongo et al. (2020) emphasised that the home visits by CHWs in WBPHCOTs build a lasting and sustainable relationships with MHCUs, families and communities. The findings affirmed that the household visitation by CHWs in the WBPHCOTs could be very crucial for the support of the service users, including the MHCUs. The implementation of mental health services by the CHWs can only be possible when there is collaboration with the mental health key stakeholders in the community.

Collaboration of WBPHCOTs with mental health key stakeholders can be enhanced by having regular meetings between the WBPHCOTs and relevant mental health internal and external stakeholders. In addition, participants recommended that the WBPHCOTs should have a resource list, so that they are knowledgeable about the available community resources and how to access them. In support, Javadi et al. (2017); D’Ambruoso et al. (2023), further stressed that strong referral routes are a crucial component of integration in community mental health, and collaboration across stakeholders is required to guarantee proper patient follow-up and treatment delivery, not just within the health system but also across other social sectors. Similarly, Mabunda et al. (2022) reported that the CHWs view partnerships with key stakeholders like families, healthcare workers, and traditional healers as an opportunity to increase access to care.

However, D’Ambruoso et al. (2023) explained that the intersectoral forums established to support the CHWs provide access to a greater variety of community referral pathways that could be utilised to connect their patients with the resources they require to succeed, thus indirectly improving mental health. The findings revealed that CHWs in the WBPHCOTs are required to have a good working relationship with the community key stakeholders to enable them to implement mental health services in their services. The training on mental health is necessary to enable the CHWs in the WBPHCOTs to implement mental health services.

Training of CHWs working in the WBPHCOTs was strongly suggested as one of the contents to be included in the guidelines for the implementation of mental health services in the WBPHCOTs. In support, Mendenhall et al. (2014); Jacobs et al. (2021) attested that the lack of training of CHWs was seen as a major challenge for the feasibility of task-sharing mental health services. Similarly, the study by Mabunda et al. (2022) revealed that the CHWs lacked the necessary abilities to provide care for individuals with mental illness, and training was proposed to fill this gap. On the other hand, Thobane et al. (2024) concluded that with proper training of CHWs, task sharing of mental health services is feasible. In addition, a more organised training programme is required to enhance the competencies of this cadre and maximise their role in the community (Windarwati et al. 2023).

In view of the above, CHWs may get ongoing training and supervision, and it’s critical to also develop local supervisors and trainers (Mabunda et al. 2022). Similar findings by Windarwati et al. (2023) assert that the CHWs with training in mental health can successfully administer mental health initiatives in the community because they possess the necessary knowledge and skills. Most significantly, Mabunda et al. (2022) further mentioned that the basic mental healthcare competencies should be included in CHW training. Therefore, it is critical that training and support for CHWs are designed to respond to the requirements of the communities they serve (Heyns et al. 2025). The findings suggest that continuous training may be provided to the CHWs in the WBPHCOTs to enable them to implement mental health services at the community level. The CHWs in the WBPHCOTs may also be involved in mental health prevention and promotion awareness campaigns to tackle the issue of stigma.

The involvement of CHWs working in the WBPHCOTs in the mental health promotion and awareness campaigns was also suggested as one of the contents to be included in the guidelines for the implementation of mental health services in the WBPHCOTs. This finding is consistent with that of Schneider et al. (2020), who explained that the CHWs could promote healthy lifestyle choices and health-seeking behaviours, and increase mental health awareness among individuals, families, and communities. In support of the above, Jacobs and Strumpher (2024) discovered that families face challenges in living with a mentally ill family member, and hence support is required in their households. Similarly, Windarwati et al. (2023) revealed that MHCUs and families have little information about the availability and access to mental health care services, as well as misconceptions about mental health as a disease caused by the devil and witchcraft.

In view of the above, Mabunda et al. (2022) discovered that CHWs in the WBPHCOTs would be able to promote awareness in the community and among families of those suffering from mental illness, hence increasing knowledge about the condition and available services.

According to Windarwati et al. (2023), the inclusion of CHWs in WBPHCOTs in the community mental health awareness activities is one strategy to improve mental healthcare, close treatment gaps and raise awareness about mental health. Similarly, Tilahun et al. (2017); Saade et al. (2023) assert that the CHWs may raise mental health awareness through mass media campaigns and/or hold mental health awareness meetings in the community to discuss issues related to mental health. The findings reveal that the CHWs in the WBPHCOTs, could be the vehicle through which the department delivers the mental health awareness messages to the community and reduce stigma attached to mental health. The CHWs in the WBPHCOTs should also ensure that the MHCUs adhere to prescribed medication and advocate for them where necessary.

The participants recommended that the CHWs in the WBPHCOTs could ‘ensure that the MHCUs adhere to the prescribed medication’ and ‘advocate for those who require assistance’ be among the content for inclusion in the guidelines. These findings are consistent with those of Mabunda et al. (2022), who mentioned that compared to community-based therapy offered by health professionals, CHW-delivered community-based therapies for individuals with mental illness in India have demonstrated higher efficacy in lowering disability and improving treatment adherence. The findings reveal that the CHWs are well positioned to visit the MHCUs at their respective homes to remind them about the importance of taking their medication as prescribed and advocate for them, given that MHCUs are one of the vulnerable groups within the community. The implementation of mental health services by the CHWs can only be possible if they receive training and support from the mental health professionals.

The importance of communication between the WBPHCOTs and the mental health professionals was identified as one of the most significant contents to be included in the guidelines. In support, Schneider et al. (2020) reported that two-way communication between the WBPHCOTs and mental health professionals enables support of the CHWs in the WBPHCOTs by the mental health professionals. This will result in improved mental health services rendered by the CHWs because they know that the support is always available when required. In addition, Matsi, Lekalakala-Mokgele and Madumo (2023) asserted that the supervision and support of CHWs by health professionals (mental health) is considered as one of the strategies to achieve universal healthcare, given that in South Africa, the WBPHCOTs’ policy and strategy are incorporated in the national health insurance policy component. The strengthening of community mental health services right through to the PHC services will enhance the work that the CHWs do at community levels because they will have an effective referral point.

The resource constraints in the PHC facilities include things like insufficient trained mental health professionals and mental health services where the CHWs would normally refer the identified cases from the community (Thobane et al. 2024). In support of the above, Mhlongo and Lutge (2019) assert that the PHC facilities’ challenges consist of shortage of mental health professionals, resources and poor infrastructure. Furthermore, Tilahun et al. (2017); Kumar et al. (2021) revealed that the majority of the CHWs they interviewed revealed that their workloads were compounded by the lack of treatment and mental health services at PHC level.

In view of the above, Susanti et al. (2025) asserted that an increase in the number of mental health professionals at PHC level who can provide mentoring and support to the CHWs in the WBPHCOTs would have a positive impact on the outreach services for mental health. In addition, Devkota et al. (2021) stressed that accessibility of health facilities and availability of comprehensive mental health services in PHC facilities could help improve the mental health services in the community and improve the referral route for the identified cases in the community. The findings reveal that for the CHWs to be effective in the implementation of mental health services, functional PHC mental health services are essential.

### Recommendations

The study has focused on the content that should be included in the development of the guidelines for implementation of mental health in the WBPHCOTs services. The policy makers could develop the strategies that will empower the CHWs in the WBPHCOTs to implement mental health services in their services. It is recommended that the policymakers facilitate the development of accredited training for CHWs, which will capacitate them to address various health programmes at community level. The CHWs should receive the necessary support and equipment from the Department of Health to enable them to implement health programmes inclusive of mental health at community level.

Future studies should also explore how the policymakers can engage the experts and the service users who might be able to assist the policymakers to develop the strategies that will enhance the implementation of mental health services by CHWs. Furthermore, a study about the perceptions of CHWs on the implementation of mental health services within their context may be undertaken in future. Additionally, South Africa is a diverse country, which then necessitates similar research conducted in a different context, for example, in rural areas.

Future research is recommended on the strengthening of PHC facilities’ mental health services to be able to receive referrals from the community level. Finally, it is recommended that future research be conducted on the support that the WBPHCOTs need from the Mental Health professionals.

### Limitations

There are several limitations to this research. Firstly, the study was only conducted in one district of Gauteng province. However, all the other four districts in the province are implementing the WBPHCOTs services in the same manner, guided by the provincial office and access to resources is the same in all five districts of the province. Future research might be necessary in another context (e.g., a different province).

Secondly, most of the participants were nurses from both the mental health team and WBPHCOTs. The researcher assumes that this is the true reflection of the health system in South Africa, where the nurses are the majority among the health professionals. However, the other health professionals who were involved in this study were given the opportunity to present their opinions. Thirdly, in terms of the WBPHCOTs, only females participated in the research study. However, the researcher assumes that the findings would have been the same since the conditions of employment are the same for all regardless of gender. Lastly, methodological limitations of the NGT. The NGT utilises a small group of participants, which may limit the opinions and consensus of other stakeholders. However, the researcher ensured that the primary stakeholders in the implementation of mental health services at community level are involved in the study.

## Conclusion

This study explored what content to include in the guidelines for the implementation of mental health services in the WBPHCOT’s services. Both NGT groups proposed seven contents or recommendations to be included: visits to MHCUs’ households, collaboration of WBPHCOTs with mental health key stakeholders, training of CHWs working in the WBPHCOTs, mental health prevention and promotion, adherence to treatment and advocacy for MHCUs, mental health professionals’ support to WBPHCOTs and strengthen community mental health services. The study will help to develop the key competencies necessary for CHWs to implement mental health services and to inform programmes and in-service training topics. The implications of this study to policymakers are that they could develop policies that empower the CHWs in the WBPHCOTs to implement the mental health services within their capabilities at the community level. Furthermore, based on the identified content to be included in the guidelines for implementation of mental health services in the WBPHCOTs, the development, validation and piloting of the guidelines may be explored in future research.
